# Male‐specific alleles in the Ryukyu drywood termite *Neotermes sugioi*

**DOI:** 10.1002/ece3.6671

**Published:** 2020-09-01

**Authors:** Ayaka Agarie, Yasushi Miyaguni, Koji Sugio, Kazuya Kobayashi

**Affiliations:** ^1^ Graduate School of Education University of the Ryukyus Nishihara Japan; ^2^ Global Education Institute University of the Ryukyus Nishihara Japan; ^3^ Hokkaido Forest Research Station Field Science Education and Research Center Kyoto University Shibecha Hokkaido Japan

**Keywords:** genetic markers, geographical variation, Isoptera, sex determination

## Abstract

Sex‐determination systems often show remarkable diversity in upstream signals, although downstream genes are broadly conserved. Therefore, the downstream genes have been investigated in various taxa, but the most upstream signals determining sex in insects have been well‐described mainly in model organisms, including fruit flies and honey bees, and not in hemimetabolous insects such as termites. Identification of sex‐linked genetic markers in termites is important to the survey of primary sex‐determination signals. Here, we report male‐specific alleles at the microsatellite locus NK12‐1 in the Ryukyu drywood termite *Neotermes sugioi* (Kalotermitidae). This study provides the third example of a genetic marker linked with sexual phenotype in termites, which is a small but important step to elucidate the evolutionary process of the sex‐determination system in termites.

## Graphical abstract

An incipient colony of *Neotermes sugioi* found from a twig. Our microsatellite analysis revealed male‐specific alleles at the microsatellite locus NK12‐1 in *N. sugioi*, which is a small but important step to elucidate the evolutionary process of the sex determination system in termites.

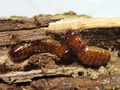

## 1 INTRODUCTION

Sex determination is one of the fundamental differentiation processes in the embryonic development of most animals. Although some of the major genes (*doublesex* and *transformer*) acting at the bottom of the sex‐determining cascade are broadly conserved, the most upstream signals in the sex‐determination cascade are remarkable diverse (Price, Egizi, & Fonseca, 2015; Suzuki, 2010; Verhulst, van de Zande, & Beukeboom, 2010). This diversity poses a major barrier to the study of the evolutionary process of sex‐determination systems, as it prevents the identification of the most upstream signals in phylogenetically distant species and makes interspecific comparisons difficult. To the best of our knowledge, the most upstream signals of sex determination in hemimetabolous insects have not been revealed, unlike in model organisms, such as fruit flies and honey bees. Thus, how the sex‐determination process evolved in Hemimetabola remains unclear.

Termites, a group of hemimetabolous insects, show a male heterogametic karyotype (Bergamaschi, Dawes‐Gromadzki, Scali, Marini, & Mantovani, 2007; Luykx, 1990). Sex‐linked genetic markers have been found in *Reticulitermes speratus* (Kolbe, 1885) (Rhinotermitidae) (Hayashi et al., 2017) and *Cavitermes tuberosus* Emerson, 1925 (Termitidae) (Fournier, Hellemans, Hanus, & Roisin, 2016), both of which show male heterozygosity and female homozygosity. These studies suggest that termites may have a common sex‐determination system. However, cytogenetic studies revealed that most species of phylogenetically derived lineages (Rhinotermitidae, Termitidae) are karyotypically uniform (2*n* = 42), while basal lineages (Kalotermitidae, Termopsidae) have more variable karyotypes (2*n* = 28–56) (Bergamaschi et al., 2007; Luykx, 1990). Although the basal termite lineages show high diversity in their karyotype, there has been no report of sex‐linked genetic markers found in these lineages.

The Ryukyu drywood termite *Neotermes sugioi* Yashiro et al., 2019, recently described as a new species (formerly *N. koshunensis*) ( Yashiro, Takematsu, Ogawa, & Matsuura, 2019), belongs to Kalotermitidae (one of the basal lineages) and distributed in the Ryukyu Islands of Japan. Identifying the association between sex‐linked genetic markers and sexual phenotypes in this species would help clarify the most upstream signals of sex determination in this species and elucidate the evolutionary process of sex determination in the termite. Here, we report that one of previously developed microsatellite loci in this species (Kobayashi & Miyaguni, 2016) is tightly linked with their sexual phenotype and has male‐specific alleles.

## 2 MATERIALS AND METHODS

We sampled 12 colonies of *N. sugioi* nesting in dead branches of living trees. Eight of them were from the Okinawa Island, Japan, and the rest from Ishigaki Island (Table S1). All the sampled branches were brought to the laboratory of Ryukyu University (Nishihara city, Okinawa Island, Japan). Then, cutting the nested branches into small blocks, all colony members, including reproductives, were collected as far as possible. The alates of each colony were separated by sex based on the morphology of abdominal sternites (Miyaguni, Sugio, & Tsuji, 2012) and preserved with ethanol until genotyping.

A total of 288 alates (12 males and females from each of the 12 colonies) were genotyped at the nine loci reported in a previous study (Kobayashi & Miyaguni, 2016). A leg of each alate was crushed in 200 μl tubes, to which 0.5 μl of proteinase K (20 mg/ml) and 100 μl of 10% Chelex solution (10 mM Tris‐HCl, 1 mM EDTA; pH 8.0) were added; the mixture was incubated at 35°C overnight. Subsequently, the mixture was boiled at 95°C for 15 min to inactivate the proteinase K. After centrifugation, the water layer was used as a template DNA. Each of the 15.2 µl reaction mixtures contained 5 pmol of each primer and 1 pmol of dyed reverse primer (Table S2), 1 μl of dNTP mix (10 mM each), 3 μl of 25 mM MgCl_2_, 3 μl of 5 × Buffer, 0.1 μl of KAPATaq EXtra DNA Polymerase (NIPPON Genetics; 5 U/μl), and 1 μl template DNA. The reactions were run according to the PCR cycle, which consisted of an initial denaturation step at 94°C for 2 min, followed by 35 cycles of 30 s at 94°C, 30 s at 60°C and 30 s at 72°C, and one step at 72°C for 1 min to complete the extension at the end. The length of PCR products was checked by SeqStudio Genetic Analyzer (Applied Biosystems) with 0.5 μl of GeneScan 600 LIZ dye Size Standard v2.0.

This species can reproduce offspring parthenogenetically by automixis with terminal fusion (Kobayashi & Miyaguni, 2016), which suggest that homozygous females at all loci may have been reproduced by parthenogenesis. Therefore, we analyzed the genotype data in two ways: with and without homozygous females at all loci.

## 3 RESULTS

**FIGURE 1:**
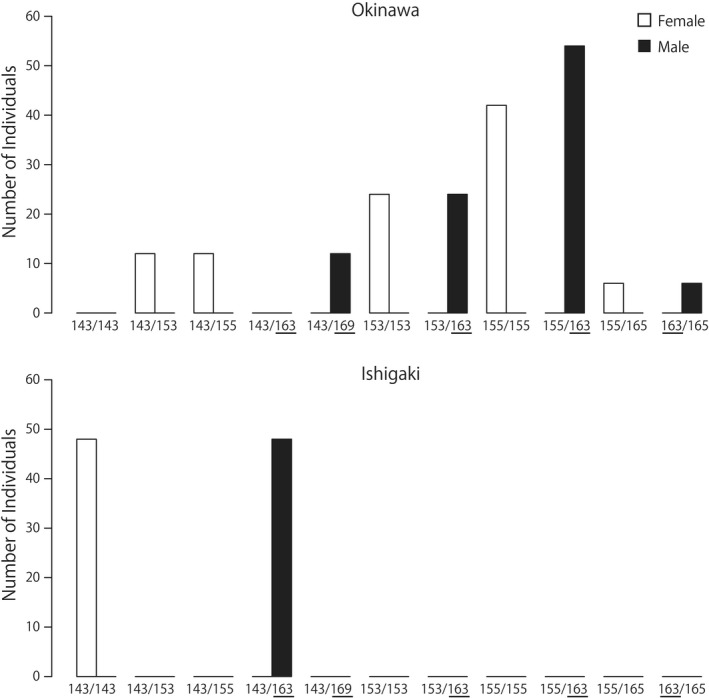
Sexual differences in the microsatellite genotype frequencies (NK12‐1) of the Ryukyu drywood termite *Neotermes sugioi* (Kalotermitidae). In each panel, filled and open bars indicate the data of male and female *N. sugioi* termites, respectively. All males have the male‐specific alleles (163 and 169 bp)

Fragment length analysis of PCR products revealed that one microsatellite locus (NK12‐1) was tightly linked with sexual phenotypes in *Neotermes sugioi* (Figure 1, Table S3). At the locus NK12‐1, all 96 males were heterozygous, but 66 of 96 females were homozygous in the Okinawa population (Fisher's exact test, *p* < .0001). In the Ishigaki population, all 48 males and 48 females were heterozygous and homozygous, respectively (Fisher's exact test, *p* < .0001). The alleles (163 and 169 bp) of NK12‐1 were detected only in males, while the other alleles (143, 153, 155, and 165 bp) were observed in both males and females. Thus, all 144 genotyped males were heterozygous for the male‐specific alleles and the shared alleles at NK12‐1 (Figure 1).

Two other microsatellites (NK14‐5 and NK8‐2) were partially linked with sexual phenotypes. At the locus NK14‐5, all 96 males and two out of 96 females in the Okinawa population were heterozygous (Fisher's exact test, *p* < .0001). In the Ishigaki population, all 48 males and 15 out of 48 females were heterozygous at this locus (Fisher's exact test, *p* < .0001). Some NK14‐5 alleles (219, 223, and 229 bp) were male‐specific, while others (209, 215, 221, and 225 bp) were found in both males and females. Individuals with the genotype 209/221 at NK14‐5 were males in the Okinawa population but females in the Ishigaki population (Figure S1). At the locus NK8‐2, 68 out of 96 males and 45 out of 96 females in the Okinawa population were heterozygous (Fisher's exact test, *p* = .0012). Especially, in a colony of Okinawa population (MY170609c2), 11 out of 12 males and one out of 12 females were heterozygous (Fisher's exact test, *p* = .0001, Table S3). However, in another colony (AG20200611c4), all 12 males were homozygous, all 12 females were heterozygous (Fisher's exact test, *p* < .0001, Table S3). In the Ishigaki population, 11 out of 48 males and 10 out of 48 females were heterozygous at NK8‐2 (Fisher's exact test, *p* = 1.0000; Figure S1).

At the other six loci, there were no significant differences in the frequency of heterozygosity between males and females in both Okinawa and Ishigaki populations (Fisher's exact test, *p* > .05; Figure S1, Tables S3 and S4). Ten out of 144 females were all homozygous at all the nine loci we genotyped. Even when these potentially parthenogenetically produced females were excluded from the analysis, the results were almost identical: males tended to be heterozygous compared with females at the three loci (*p* < .0001 for NK12‐1 and NK14‐5 in both populations, *p* = .0065 for NK8‐2 in Okinawa population, *p* > .05 for others; Table S4).

## 4 DISCUSSION

Our microsatellite analysis revealed male‐specific alleles in the locus NK12‐1 of *N. sugioi* (Figure 1), suggesting that this locus may be located closer to the most upstream sex‐determination signals. Moreover, the number of male‐specific alleles was less than that of alleles shared between sexes, which might be arising from difference between the Y and X chromosomes. The Y chromosome is transmitted from male to male offspring only, while the X chromosome is transmitted from female to male and female offspring and from male to female offspring. Therefore, compared to Y chromosome, the mutation rate per generation will be high on the X chromosome and the variation is likely to be maintained in the population.

Contrary to the clear link of NK12‐1, those of NK14‐5 and NK8‐2 are obscure. This incomplete linkage can be explained by the distance from sex‐determining gene. These loci might be located on the sex chromosome but relatively far from the sex‐determining gene compared with NK12‐1, and recombination broke the linkage between these two loci and the most upstream signal of sex determination in some populations. Interestingly, in a colony AG20200611c4 at NK8‐2, all males were homozygous (148/148) and all females were heterozygous (148/154). This pattern can be explained by a heterozygous father (X linked 154 bp and Y linked 148 bp) and a homozygous mother (X linked 148 bp). The heterozygous males (148/154) were actually found in a colony AG20200611c2, where the allele 148 bp was found only in males (Table S3). Thus, this result is also consistent with the hypothesis that this locus is linked to the sex‐determination gene but the link is easily broken by recombination due to its long distance from the sex‐determination gene.

Another potential mechanism explaining weak linkage of NK14‐5 and NK8‐2 is multi‐chromosome translocation complexes in male meiosis. In males of some termites, multiple autosomes are translocated with sex chromosomes as if they were linked, and inherited in the same way as the sex chromosomes, while in females, chromosomes are translocated independently (Bergamaschi et al., 2007; Luykx, 1990; Syren & Luykx, 1977). This phenomenon has been confirmed in *N. fulvescens* (Martins & Mesa, 1995), so it may occur in *N. sugioi*. Thus, relatively weakly linked loci (NK14‐5 and NK8‐2) may exist on chromosomes that are not sex chromosomes but on sex‐linked autosomes. In the termites *Incisitermes schwarzi* and *Kalotermes approximatus* (Kalotermitidae), number of chromosomes in the translocation complex varies among the population (Luykx, 1987; Syren & Luykx, 1977, 1981). Such geographic difference might explain the difference in the linkage strength of these slightly linked loci in *N. sugioi*. To clarify these points, karyological studies for multiple populations of *N. sugioi* are required.

In hemimetabolous insects such as termites, the most upstream signal of sex determination is unknown. This study is the first report of sex‐linked genetic markers in Kalotermitidae. Although the sex‐determination mechanism has not been elucidated in termites, sex‐linked genetic markers were reported from two termite species belonging to more derived lineages than *N. sugioi* (Fournier et al., 2016; Hayashi et al., 2017). In these two termites and in ours, males are heterozygous and females are homozygous, which supports a widely conserved XY sex‐determination system in termites. Recent remarkable advances in high‐throughput sequencing technology have made it easier to obtain draft genomes even for nonmodel organisms. Mapping of these sex‐linked genetic markers on draft genomes obtained from the target species may help clarify the location of the most upstream signals on the genomes of termites. Moreover, in the three species reported in ours and previous studies (Fournier et al., 2016; Hayashi et al., 2017), a comparison of genes located around the sex‐linked markers may reveal how sex‐determination systems evolved in termites, which would be an important stepping stone in clarifying the evolutionary history of sex determination in insects.

## CONFLICT OF INTERESTS

The authors declare no competing financial interests.

## AUTHOR CONTRIBUTION

**Ayaka Agarie:** Data curation (equal); Formal analysis (equal); Investigation (equal); Resources (equal); Validation (equal); Writing‐review & editing (equal). **Yasushi Miyaguni:** Resources (equal); Validation (equal); Writing‐review & editing (equal). **Koji Sugio:** Conceptualization (equal); Methodology (equal); Supervision (equal); Validation (equal); Writing‐review & editing (equal). **Kazuya Kobayashi:** Data curation (equal); Formal analysis (equal); Funding acquisition (equal); Investigation (equal); Resources (equal); Validation (equal); Visualization (equal); Writing‐original draft (equal); Writing‐review & editing (equal).

## Supporting information

Figure S1

Tables S1–S4

## Data Availability

Sampling locations and microsatellite genotypes: Supporting information (Figure S1 and Tables S1–S4).
